# Non-coding genome in nail-patella syndrome: Genetic diagnosis as a guide for personalized follow-up

**DOI:** 10.1038/s41431-026-02062-5

**Published:** 2026-03-03

**Authors:** Perrine Brunelle, Anne-Sophie Jourdain, Fabienne Escande, Séverine Audebert-Bellanger, Sonia Bouquillon, Valérie Cormier-Daire, Alexandra Desdoits, Didier Lacombe, Arnaud Molin, Nicolas Gruchy, Renaud Touraine, Julien Van Gils, Jamal Ghoumid, Luc Thomes, Florence Petit

**Affiliations:** 1 Univ. Lille, CHU Lille, ULR7364 RADEME, FHU G4 Génomique, F-59000 Lille, France; 2 CHU Brest, Service de génétique clinique, Centre de compétences des maladies osseuses constitutionnelles (filière OSCAR), F-29600 Brest, France; 3 CHU Lille, Institut de génétique médicale, F-59000 Lille, France; 4 Université Paris Cité, Centre de référence pour les maladies osseuses constitutionnelles, Institut Imagine, INSERM UMR 1163, Hôpital Necker-Enfants Malades, Paris, France; 5 CHU Caen Normandie, Service de Chirurgie pédiatrique et rhumatologie pédiatrique, Centre de compétence des maladies osseuses constitutionnelles (filière OSCAR), F-14000 CAEN, France; 6 Service de génétique médicale, CHU Bordeaux ; MRGM INSERM U1211, Université de Bordeaux, Bordeaux, France; 7 Université de Caen Normandie, Normandie Univ, BIOTARGEN UR7450, CHU Caen Normandie, Service de Génétique, Centre de référence des maladies rares du calcium et du phosphate (filière OSCAR), F-14000 Caen, France; 8 Service de génétique clinique chromosomique et moléculaire, CHU de Saint-Etienne, Saint-Etienne, France; 9 Laboratoire AURAGEN, PFMG2025 Lyon, France

**Keywords:** Diseases, Genetics research, Genetic testing

## Abstract

Limb malformations are paradigmatic of altered gene regulation in human disease. Nail-Patella Syndrome (NPS) is a rare condition characterized mainly by skeletal defects, glomerulonephritis and glaucoma, with variable expressivity. NPS is caused by the haploinsufficiency or loss-of-function of *LMX1B*, which encodes a transcription factor involved in limb dorsalization, in the renal glomerular filtration barrier and the anterior segment of the eye. The dorsal expression of *LMX1B* in the developing limbs is under the control of *LMX1B* autoregulatory modules (LARMs), which are non-coding *cis*-regulatory elements (CREs) with a limb-specific enhancer activity. Here, we describe the regulatory landscape and report regulatory anomalies at the *LMX1B* locus in four families, including the deletion of a CRE, two structural variations disrupting the CRE-promoter interaction, and a 5’UTR variant causing an upstream open reading frame (ORF). Molecular mechanisms involving the non-coding genome can have a tissue-specific impact on gene expression, resulting in incomplete forms of the syndrome, and sometimes modifying its classical mode of inheritance. While approximately 95% of individuals with NPS carry pathogenic variants in the coding regions of *LMX1B*, non-coding alterations explain the remaining cases. This work highlights the importance of genomic diagnosis (gene ORF versus CRE alteration) for precision medicine and genetic counselling in rare diseases.

## Introduction

Nail-Patella syndrome (NPS, MIM#161200) is a rare autosomal dominant condition characterized by variable expression of skeletal anomalies related to an abnormal dorsalization of the limbs, such as absent or hypoplastic patella, radial head dysplasia, iliac horns and dystrophic nails (longitudinal ridging, triangular lunula, koilonychia, anonychia). This association of features is recognizable and iliac horns are even pathognomonic for the condition. Affected patients may also have kidney and/or ocular anomalies, leading to frequent follow ups and illustrating the importance of diagnosing this rare disease. Kidney anomalies have been documented in 20–50% of cases, their prevalence increasing with age [1, 2]. This is a glomerular nephropathy responsible for proteinuria, with or without hematuria, which may remain asymptomatic or progress to a nephrotic syndrome or even chronic and end-stage renal failure. Ocular anomalies consist of isolated ocular hypertension or open-angle glaucoma, which prevalence has been little studied (between 20% and 33% depending on the series) [3].

NPS is mostly due to pathogenic variants in *LMX1B* (*LIM homeobox transcription factor 1 beta*, MIM*602575), encoding a LIM-homeobox transcription factor, leading to haploinsufficiency or loss-of-function [4–6]. In this condition, the molecular diagnostic yield is high, up to 90-95% of NPS-affected individuals carrying an alteration of the *LMX1B* gene [1].

In 2017, using Lmx1b ChIP-seq on mouse limbs, Haro et al. identified two autoregulatory modules for *Lmx1b*, directing its expression in the dorsal part of the developing limbs [7]. These non-coding regions, named LARM1 and LARM2 (*Lmx1b* AutoRegulatory Modules), are *cis*-regulatory elements (CREs) located around 60 kb upstream of the promoter. The mouse homozygous knock-out for LARM1/2 shows absence of Lmx1b expression in limbs and isolated skeletal anomalies compared to the *Lmx1b* knock-out who recapitulates typical Nail-Patella syndrome including ocular and renal involvement [8]. So far, alterations of these limb-specific enhancers have been reported in 3 unrelated families affected with skeletal anomalies typical of NPS [8, 9]. Consistently with the mouse model, these individuals do not show any ocular or renal involvement. Two families are carriers of heterozygous deletions comprising the entire LARM2 but respecting LARM1, segregating in a dominant autosomal mode of inheritance [8, 9]. In a sporadic consanguineous case, homozygous rare SNVs in LARM2 were responsible for the loss of its activity and *LMX1B* haploinsufficiency in a recessive autosomal mode of inheritance [8], illustrating that enhanceropathies may complicate genetic counselling and that the molecular diagnosis is important for both the genetic counselling and the adaptation of individual follow-up.

Here we report on four novel examples of *LMX1B* deregulation in NPS. We review the molecular mechanisms that may be involved in the pathogenesis and guide the personalized follow-up. With that recent knowledge, the molecular diagnostic yield reaches close to 100% in NPS.

## Subjects and methods

### Subjects

Four individuals affected with Nail-Patella syndrome were recruited through the French reference network for rare diseases. In all cases, pathogenic/likely pathogenic variants were previously ruled out in the *LMX1B* coding regions through gene panel sequencing as described before [10]. Patients and/or parents gave their consent for high-throughput sequencing on a diagnosis basis.

### High-throughput sequencing

For patient 1, *LMX1B* coding regions and flanking introns and LARM1/2 non-coding regions were studied by targeted high-throughput sequencing on DNA extracted from blood samples, as described before [10]. For patients 2, 3 and 4, trio genome sequencing was performed thanks to the 2025 French Genomic Medicine Initiative [11].

### Real-time quantitative Polymerase Chain Reaction (qPCR)

For patient 1, the LARM1/2 deletion identified by high-throughput sequencing was refined by qPCR before precise mapping of the breakpoints by Sanger sequencing. Real-time qPCR was performed using SYBR Green technology (Applied Biosystems®, Saint Aubin, France) and several primer pairs available in Supplementary Table 1. Quantification of the target sequences was normalized to an assay from RPH Polymerase (NR_002312), and the relative copy number was determined on the basis of the comparative ΔΔCt method using a normal control DNA as the calibrator.

### Sanger sequencing

All genomic variants described were confirmed by Sanger sequencing using the AmpliTaq Gold™ DNA Polymerase (Thermo Fischer Scientific). Amplicon fragments were sequenced using the ABI Prism 3730XL Genetic Analyzer (Applied Biosystems, Courtaboeuf, France). Primers are provided in the Supplementary Table 1.

### In silico analyses

Publicly available Hi-C datasets were analyzed to assess chromatin contacts between candidate CREs and the *LMX1B* promoter. We focused on the topologically associated domain (TAD) containing *LMX1B* (chr9:125,400,000-126,850,000(hg38)), defined by high chromatin interactions from in situ Hi-C and Micro-C XL experiments on the H1-hESC (embryonic stem cells) and HFFc6 (foreskin fibroblasts) cell lines [12].

Hi-C data from H1-hESCs (dataset 4DNFIALNLR78 [13], 4D Nucleome Data Portal [14, 15]) were used to model chromatin architecture at a 5 kb resolution. The refined three-dimensional conformation of this region was reconstructed using the Spring model software (https://spring-model.mini.pw.edu.pl/), employing a self-avoiding random walk algorithm with a stiffness parameter of 1 and using the 5% strongest contacts as structural constraints. The resulting 3D structure was visualized, and genomic elements of interest were annotated using UCSF ChimeraX version 1.10.1.

We used the ReMap Atlas of regulatory regions [16], which provides a large-scale integrative analysis of all public ChIP-seq data for transcriptional regulators from GEO, ArrayExpress, and ENCODE, to identify peaks in specific cell lines or tissues compared to all others. We focused on the data from HEK293 cell lines, kidney and retina tissue, where *LMX1B* is expressed. These peaks were combined with vertebrate sequence conservation and chromatin marks data (DNAse I hypersensitivity, H3K27ac, H3K4me1, H3K4me3) to identify enriched regions suggesting CREs that may regulate gene expression in the developing kidney or retina.

## Results

### Patient 1

Patient 1 is a 22-year-old female born from unrelated parents. She presents with dystrophic hand nails, radial head dysplasia with posterior subluxation and elbow mobility defect, hypoplastic patellae and iliac horns, typical for NPS syndrome. Ophthalmological examination and renal function are normal, with no proteinuria. Several relatives on the maternal side are affected with NPS syndrome (Supplementary Fig. 1). The mother presents with dystrophic hand nails, hypoplastic patellae and iliac horns, but no ocular or renal features. Clinical data on the other relatives is limited.

*LMX1B* and LARM1/2 were studied by targeted high-throughput sequencing, showing a heterozygous deletion comprising LARM1 and LARM2 but respecting the *LMX1B* gene. The breakpoints were characterized by qPCR (Supplementary Table 1) and sanger sequencing, mapping a 47 kb non-coding deletion (NC_000009.12:g.126528370_126575687delinsGTG) (Supplementary Fig. 1).

### Patient 2

Patient 2 is a 16-year-old male born from healthy and unrelated parents, with no particular familial history. He presents with dystrophic hand nails, radial head dysplasia, patella dysplasia and iliac horns, typical for NPS syndrome. Ophthalmological examination (intraocular pressure) and renal function (blood: urea 0.24 g/L, creatinine 8 mg/L, creatinine clearance CKD-EPI 132 ml/min/1,73m^2^; urine: creatinine 1527 mg/L, albumin 9 mg/L, albumin/creatinine=6 mg/g, absence of hematuria) are normal. *LMX1B* and LARM1/2 were studied by targeted high-throughput sequencing, showing no pathogenic / likely pathogenic single nucleotide or copy number variant. Trio genome sequencing was carried out on DNA extracted from blood samples thanks to the 2025 French Genomic Medicine Initiative. Examination of the *LMX1B* locus using IGV browser showed split-reads, unmasking a reciprocal translocation t(9;16)(q33.3;q22.1) that occurred de novo. The 9q33.3 breakpoint (chr9:126,564,799(hg38)) was located in the non-coding region in between *LMX1B* and LARM1/2, therefore preventing the physical interaction between the limb-specific enhancers and the promoter. The 16q22.1 breakpoint (chr16:68,844,382(hg38)) interrupted the *TANGO6* gene, a gene that is not related to a human disease so far (MIM*620188). This structural rearrangement was confirmed by karyotyping on leucocytes culture and the breakpoints were confirmed by Sanger sequencing (Supplementary Fig. 2).

### Patient 3

Patient 3 is a 16-year-old female born from healthy and unrelated parents, with no particular familial history. She presents with dystrophic hand nails, bilateral patellar hypoplasia and dislocation with genu recurvatum and iliac horns, typical for NPS syndrome. Ophthalmological examination and renal function are normal, with no proteinuria. *LMX1B* and LARM1/2 were studied by targeted high-throughput sequencing, showing no pathogenic / likely pathogenic single nucleotide or copy number variant. Trio genome sequencing was carried out on DNA extracted from blood samples thanks to the 2025 French Genomic Medicine Initiative. Examination of the *LMX1B* locus using IGV browser showed split-reads, unmasking a reciprocal translocation t(5;9)(q12.3;q33.3) that occurred de novo. The 9q33.3 breakpoint (chr9:126,546,031(hg38)) was located in the non-coding region in between *LMX1B* and LARM1/2, therefore preventing the physical interaction between the limb-specific enhancers and the promoter. The 5q breakpoint (chr5:67,184,749(hg38)) interrupted the *CD180* gene, a gene that is not related to a human disease so far (MIM*602226). This structural rearrangement was confirmed by karyotyping on leucocytes culture and the breakpoints were confirmed by Sanger sequencing (Supplementary Fig. 3).

### Patient 4

Patient 4 is a 44-year-old female born from healthy and unrelated parents. She presents with dystrophic hand nails and bilateral patellar hypoplasia, typical for NPS syndrome. Ophthalmological examination and renal function (blood: urea 0.26 g/L, creatinine 5.9 mg/L, creatinine clearance CKD-EPI 125.5 ml/min/1,73m^2^; urine: protein 0.07 g/24 h, absence of hematuria) are normal. She has an affected son presenting with dystrophic hand nails. At 8 years, his ophthalmological examination and renal function (blood: urea 0.26 g/L, creatinine 4 mg/L, creatinine clearance CKD-EPI 351.2 ml/min/1,73m^2^; urine: protein 0.06 g/24 h, absence of hematuria) are normal (Supplementary Fig. 4). Trio genome sequencing in Patient 4 and her unaffected parents was carried out on DNA extracted from blood samples thanks to the 2025 French Genomic Medicine Initiative. The analysis revealed a heterozygous pathogenic variant in *LMX1B* 5’UTR that occurred de novo: NC_000009.12:g.126614224 G > A; NM_001174147.2:c.-226G > A; p.?. Sanger sequencing confirmed the presence of the heterozygous variant in Patient 4 and its absence in both unaffected parents, and showed that her affected son is also heterozygous (Supplementary Fig. 4). This variant is absent from population databases and is predicted to create an upstream ORF and premature stop codon by the MORFEE bioinformatic tool [17]. It has already been described in one large NPS family and functional testing demonstrated impaired gene expression at post transcriptional level leading to *LMX1B* haploinsufficiency [18].

### LMX1B regulatory landscape

While *cis*-regulatory elements controlling *LMX1B* expression in the limb, LARM1 and LARM2, have been precisely characterized [7, 8], little is known on CREs active in the kidney or the eye, two other expression territories related to phenotypic features in Nail-Patella syndrome.

**Fig. 1:**
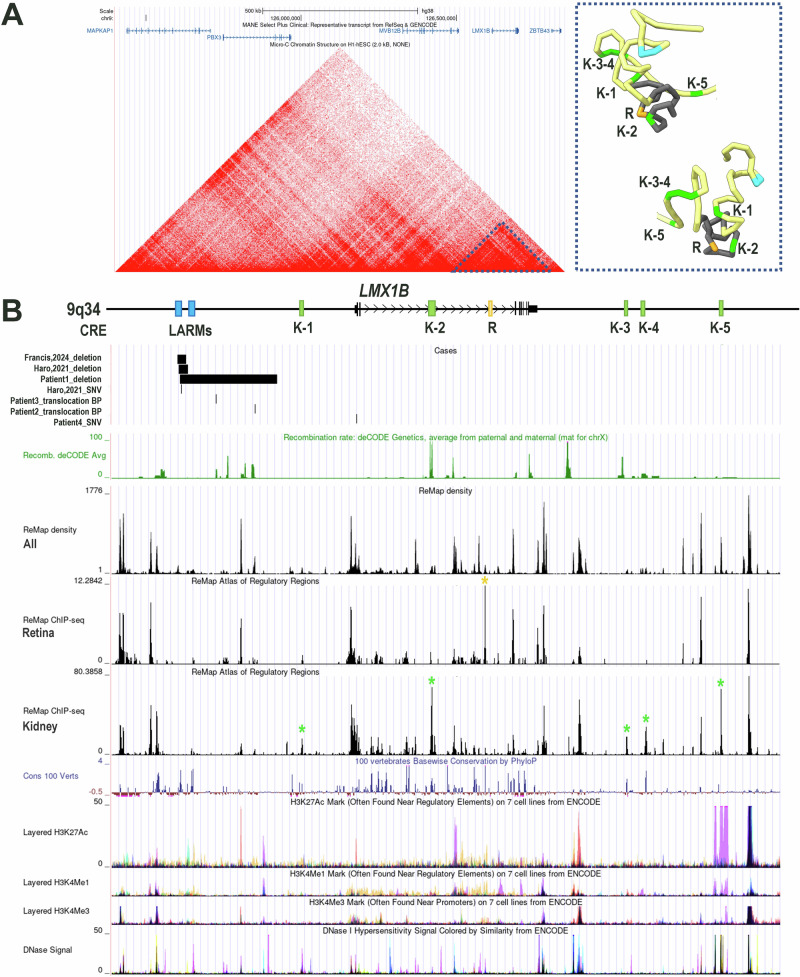
*LMX1B* regulatory landscape and regulatory anomalies. **A** Left: Micro-C chromatin structure on H1-hESC showing that significant interactions are confined to the *LMX1B* sub-TAD (chr9:126,480,000–126,850,000(hg38)), highlighted by a dashed triangle. Right: Modeling of the chromatin interactions inside the *LMX1B* sub-TAD. Dark grey: *LMX1B* gene; Blue: LARMs; Green: Candidate *LMX1B* CREs in the kidney; Orange: Candidate *LMX1B* CRE in the retina. **B** Genomic variants affecting *LMX1B* regulation. “Cases” track: location and nature of *LMX1B* regulatory variants and translocation breakpoints (BP) reported in this series and in the literature. LARM: *LMX1B* limb autoregulatory modules; K1-5 and green *: candidate *LMX1B* CREs in the kidney; R and orange *: candidate *LMX1B* CRE in the retina.

Examination of Hi-C and Micro-C XL interaction tracks from the UCSC Genome Browser (http://genome.ucsc.edu) [19] revealed that significant interactions were confined to the *LMX1B* sub-TAD (chr9:126,480,000–126,850,000(hg38)), with minimal contact observed across adjacent sub-TAD boundaries (Fig. 1A). Therefore, subsequent analyses were restricted to this sub-TAD.

Combining available experimental and vertebral conservation data, we identified several enriched regions comprised in the *LMX1B* sub-TAD that could contain kidney-specific or eye-specific CREs (Fig. 1B, Supplementary Table 2). Six candidate CREs have been selected because of ChIP-seq peaks enrichment in HEK293 cell lines and kidney tissue or retina tissue, relative to all cell lines and tissues available on the ReMap Atlas of regulatory regions. Modeling of chromatin architecture using Hi-C data from H1-hESCs shows that 5 of these candidate tissue-specific CREs may in fact interact with the *LMX1B* promoter (K-1, K-2, K-3, K-4 and R) while no interaction is predicted for the one of them (K-5) (Fig. 1A).

Apart from Patient 4 who carries a SNV in the 5’UTR of *LMX1B*, in all patients from this report and the literature with Nail-Patella syndrome due to non-coding deletion (P1 and [8, 9]), non-coding SNVs [8] or structural variations (P2 and P3), only limb-specific CREs are disrupted (Fig. 1B). These cases present with a skeletal-only phenotype. The absence of kidney or ocular features may be related to preserved *LMX1B* expression in these tissues. This hypothesis is supported by the location of predicted CREs, which are unaffected by the structural variations.

## Discussion

**Fig. 2:**
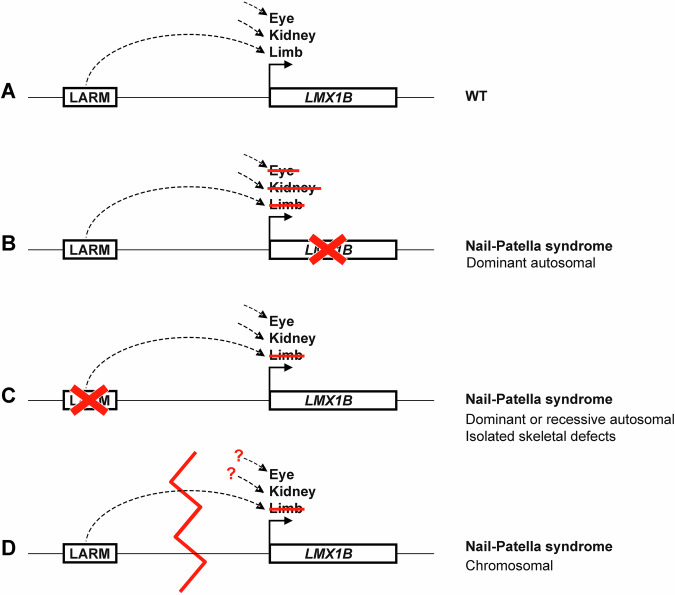
Summary of molecular mechanisms involved in Nail-Patella syndrome. **A** Wild-type (WT) locus (not at scale). **B** Typical Nail-Patella syndrome due to gene defect. **C** Skeletal-only Nail-Patella syndrome due to LARM (*LMX1B* Limb AutoRegulatory Modules) defect. **D** Nail-Patella syndrome due to structural variation of the genome (broken red line) disrupting the interaction between enhancer and promoter (dashed line).

Since our Nail-Patella syndrome series publication in 2016 [1], the knowledge on *LMX1B* regulation in dorsal limbs has significantly improved [7, 8], breaking the diagnostic deadlocks in this condition. In our experience, the diagnostic yield reaches now 100% in NPS, with nearly 95% of cases harboring pathogenic/likely pathogenic variants in coding or flanking intronic regions, while the remaining cases are resolved by the identification of regulatory anomalies affecting the non-coding genome. Here we report four examples of *LMX1B* regulation defects due to deletion of *cis*-regulatory elements, disruption of enhancer-promoter interaction, or 5’UTR variant responsible for an upstream ORF. Molecular mechanisms involved in Nail-Patella syndrome are summarized in Fig. 2.

Variants in the non-coding genome are challenging to interpret. So far in the ClinVar database, only one pathogenic variant is reported in *LMX1B* UTRs, while almost one hundred variants of uncertain significance are reported, stressing the need for powerful in silico tools and functional tests. The MORFEE bioinformatic tool [17] was efficient to predict an upstream ORF for the 5’UTR variant identified in Patient 4 (NM_001174147.2:c.-226G > A). This variant was already reported by Cappato et al. in an unrelated NPS family. In another family, the authors reported an additional 5’UTR variant (NM_001174147.2:c.-174C > T) and demonstrated that both variants were pathogenic and responsible for *LMX1B* haploinsufficiency, using functional tests in vitro [18].

Regulatory anomalies may change the mode of inheritance, and isolate pleiotropic features of a syndromic condition in accordance with tissue-specificity of *cis*-regulatory elements (CREs). NPS due to monoallelic or biallelic alteration in the limb-specific enhancers (LARM1 and/or 2) manifests as isolated skeletal defects of autosomal dominant or recessive mode of inheritance. This pathophysiological mechanism identified in Patient 1’s family, has been reported previously in only 3 observations [8, 9]. While more case descriptions may be needed to confirm this statement, patients with LARM alterations may not be at risk for kidney or ocular features of NPS, in line with the skeletal-only phenotype of the Larm knock-out mouse model [8]. This example demonstrates the importance of the molecular diagnosis for precision medicine and genetic counselling in rare diseases.

Characterizing the *LMX1B* regulatory landscape could bring insights in other tissue-specific CREs that may be involved in human disease. For instance, the alteration of tissue-specific CREs of *LMX1B* may be responsible for isolated kidney or ocular disease. In that regards, we identified several candidate CREs that could be investigated by functional studies such as in vivo reporter assays, and subsequently screened in affected patients with glomerular nephritis or glaucoma (Fig. 1B, Supplementary Table 2). One limitation is the lack of data on the specific cells related to these phenotypes (e.g. glomerular, ciliary body, trabecular meshwork cells). We used HEK293 cells, whole kidney tissue and retinal cells to identify these tissue-specific candidate CREs, but the regulatory landscape may differ in more relevant cells.

In two sporadic patients, we identified reciprocal translocations with 9q33.3 breakpoint in the non-coding region between *LMX1B* promoter and limb-specific enhancers, thereby disrupting the enhancer-promoter interaction. Although the two translocation breakpoints are 19 kb apart, they appear to be located within a recombination hotspot, suggesting that this pathomechanism for NPS may recur (Fig. 1B, Recombination deCODE average track [20]). In both cases, no kidney nor ocular features are observed so far, but both patients are young and those features are not fully penetrant. While candidate kidney- and ocular-specific CREs remain in their normal location and therefore should still regulate *LMX1B* in these tissues, renal and ocular follow-up is recommended in these patients according to our current knowledge. Again, the comprehensive characterization of the *LMX1B* regulatory landscape would help to provide personalized follow-up in these cases, depending on the location of the tissue-specific CREs relative to the chromosomal breakpoints.

The alteration of non-coding genome in human disease is a rapidly evolving field of knowledge, and limb malformations are paradigmatic for these mechanisms [21], probably because the complexity of limb morphogenesis and multiplicity of signaling pathways involved make it particularly sensitive to regulatory defects. As with *LMX1B* and Nail-Patella syndrome, regulatory defects have been reported in other limb malformations. For instance, Acheiropody (MIM#200500), a rare disease where the four limbs are truncated, is due to biallelic loss-of-function of the limb-specific enhancer of *SHH* (*sonic hedgehog*, MIM*600725), either because of complete deletion [22] or because of structural variations altering the enhancer-promoter interaction [23]. Other examples are tissue-specific deregulation of *DLX5* (*distal-less homebox*, MIM*600028) or *SOX9* (*SRY-box 9*, MIM*608160) genes, due to enhancer deletions or structural variation breakpoints disrupting the enhancer-promoter interactions [24, 25]. Other than limb malformations, these mechanisms are also being described in several human diseases, opening a large field of discovery in isolated malformations.

In the era of routine diagnostic genome sequencing, these examples highlight the need for systematic detection and curation of structural variations and illustrate the importance of the clinical-biological expertise and cooperation for data interpretation, particularly for non-coding genome alterations. We emphasize the need for functional characterization of tissue-specific CREs in order to guide the personalized follow-up in patients with regulatory alterations.

## Supplementary information

Supplementary data

## Data Availability

All data presented in this article are available to qualified researchers upon request. Deletion identified in patient 1 is reported into the Decipher database ID#570100 (https://www.deciphergenomics.org/), variant identified in patient 4 is reported into the ClinVar database (https://www.ncbi.nlm.nih.gov/clinvar/, accession: VCV004292043.1).
