# Supplementary material for: Non-coding genome in nail-patella syndrome: Genetic diagnosis as a guide for personalized follow-up
Source: Eur J Hum Genet. 2026 Mar 3;34(5):597–602. doi: 10.1038/s41431-026-02062-5 (PMC13172535; doi:10.1038/s41431-026-02062-5)
Supplement: Supplementary file 1 — Supplementary data [file 41431_2026_2062_MOESM1_ESM.docx]

**Supplementary Data**

**Supplementary Table 1**

| **Primer sequence 5’>3’** | **Objective** | **Results** |
| --- | --- | --- |
| Forward: GGTCCCAACCAGAATGCACC  Reverse: TTTCTCGACCTTTGGGGTGA | Mapping deletion breakpoints (Patient 1) by qPCR | Non deleted |
| Forward: TAATCTCCATAAAACAGACCCC  Reverse: CATGACTTTCAGTGGGTCCT | Mapping deletion breakpoints (Patient 1) by qPCR | Deleted |
| Forward: GCAGAAAAGGGCTGGTAACG  Reverse: CACAGGGAGAACCTTCCAGG | Mapping deletion breakpoints (Patient 1) by qPCR | Deleted |
| Forward: TGGGTCGAGATGGGGAGATT  Reverse: AGGTGCTGAGCAACCCTTTT | Mapping deletion breakpoints (Patient 1) by qPCR | Non deleted |
| Forward: CCAGGCACTCTGCATGTTTG Reverse: TTTCTCGACCTTTGGGGTGA | Mapping deletion breakpoints (Patient 1) by Sanger sequencing | NC_000009.12:g.126528370_126575687delinsGTG |
| Forward: TCCAGGTCATGCCCCTCC  Reverse: GCCGTTAGAAGACAAGTGACC | Mapping translocation breakpoints (Patient 2) by Sanger sequencing | chr9:126,564,799(hg38) |
| Forward: GGCTGGAGGCTTCCAAATCT  Reverse: TAACTTGCCCGGGATGACAC | Mapping translocation breakpoints (Patient 3) by Sanger sequencing | chr9:126,546,031(hg38) |
| Forward: GAAGTTTCGGGAGGTGAGGC  Reverse: TCCAACATCTTGGCGCAGTC | Familial segregation of the *LMX1B* 5’UTR variant (Patient 4 and relatives) by Sanger sequencing | Variant occurred *de novo* in patient 4 and is transmitted to the affected son. |

**Supplementary Table 2**

| **Candidate CRE** | **Position (hg38)** | **Binding proteins according to ChIP-seq data from ReMap Atlas of regulatory regions** |
| --- | --- | --- |
| Kidney_1 | chr9:126587270-126588462 | TRIM28, GLIS1, ZNF273, BRD4, MAZ, ZBTB12, PATZ1, ZBTB6, SETDB1, ZSCAN30, SP2, OSR2, ZNF398, MAZ, ZNF692, ZNF281, ZBTB8A, ZXDB, ZNF479, ZNF770, RAD21, FEZF1, ZNF257, ZNF711 |
| Kidney_2 | chr9:126650026-126653044 | ZBTB48, MAZ, ZNF41, GLIS1, NFKB1, ZNF366, KLF17, ZFX, WT1, ZNF341, ZBTB42, ZEB2, SP2, ZNF335, PHIP, ZNF770, OGG1, ZNF530, TRIM28, IKZF3, TFIIIC, NCAPH2, TBL1X, ZXDB, ZNF528, ZBTB8A, ZNF843, ZNF692, PRDM4, RAD21, ZFP69B, PRDM6, ZBTB12, FEZF1, ZNF423, ZNF76, KLF10, ZNF629, KLF5, ZNF580, OSR2, HIC1, ZNF660, KLF16, ZNF24, ZNF561, ZBTB44, ZNF35, BCL11A, ZNF596, ZNF189, INSM2, BCL11B, ZNF213, ZSCAN30, ZNF639, ZNF394, KLF1, ZNF10, ZNF554, ZBTB20, ZNF2, HOXA9, ZNF574, ZSCAN23, ZNF558, ZNF391, ZNF467, ZBTB21, KLF8, IRF2BP2, ZNF324, TSHZ1, ATF2, KLF9, ZNF610, MZF1, ZNF449, ZBTB6, SP3, ZSCAN22, ZFP64, SP1, PATZ1, SP4, ZSCAN16, PKNOX1, ZIM3, IKZF3, GLIS2, GLIS1, PATZ1, TCF7L2, MAZ, KLF5, ZNF398, OSR2, SP7, KLF9, ZNF366, ZEB2, ZBTB8A, KLF1, ZNF629 |
| Kidney_3 | chr9:126745261-126746108 | ZNF398, ZNF366, ZNF341, ZNF561, SP7, ZNF467, IKZF3, GLIS1, SCRT1, ZBTB8A, ZNF18, PRDM6, FEZF1, ZNF189, ZBTB44, ZFP64, OSR2, NUP98, HOXA9, ZNF580, ZEB2, ZSCAN30, ZNF35, HOXA9, ZNF695, TEAD4, ZFP69B, KLF5, SP4 |
| Kidney_4 | chr9:126754324-126755911 | PHIP, ZNF528, ZNF449, GLIS1, ZNF189, SCRT1, ZNF335, ZNF423, ZNF341, ZNF674, IKZF3, ZEB2, ZXDB, ZNF121, FEZF1, PATZ1, IRF2BP2, ZNF843, ZNF770, ZNF10, ZNF561, BCL11B, NUP98, HOXA9, ZNF711, ZSCAN30, OGG1, ZBTB8A, KLF16, PKNOX1, ZFP64, ZFX, RELA, SUZ12, SP2, ZNF574, ZBTB48, ZBTB20, PRDM6, PRDM4, ZBTB6, ZBTB42, ZNF394, ZNF778, ZNF175, NFKB1, ZNF283, KLF9 |
| Kidney_5 | chr9:126791500-126792597 | PRDM6, TEAD1, ZNF184, ZNF366, BCL11B, YY1AP1, MZF1, OSR2, ZNF561, ZNF18, TSHZ1, SMC3, IRF2BP2, ZBTB21, ZNF843, ZXDB, HIC1, PRDM4, ZNF391, PATZ1, ZNF554, ZBTB44, ZNF547, SCRT1, SP2, FEZF1, PRDM1, ZNF317, FOXA1, KLF1, IKZF3, GLI2, GFI1B, ZNF324, ZFP69B, KLF5, ZNF341, ZNF528, ZNF596, GLIS2, ZNF263, NFKB1, ZNF121, KLF17, GLIS1 |
| Retina | chr9:126676549-126677343 | RORB, CTCF, CRX, OTX2, BRD4, CRX, NRL, MEF2D |

CRE: candidate *cis*-regulatory region;

**Supplementary Figure 1**


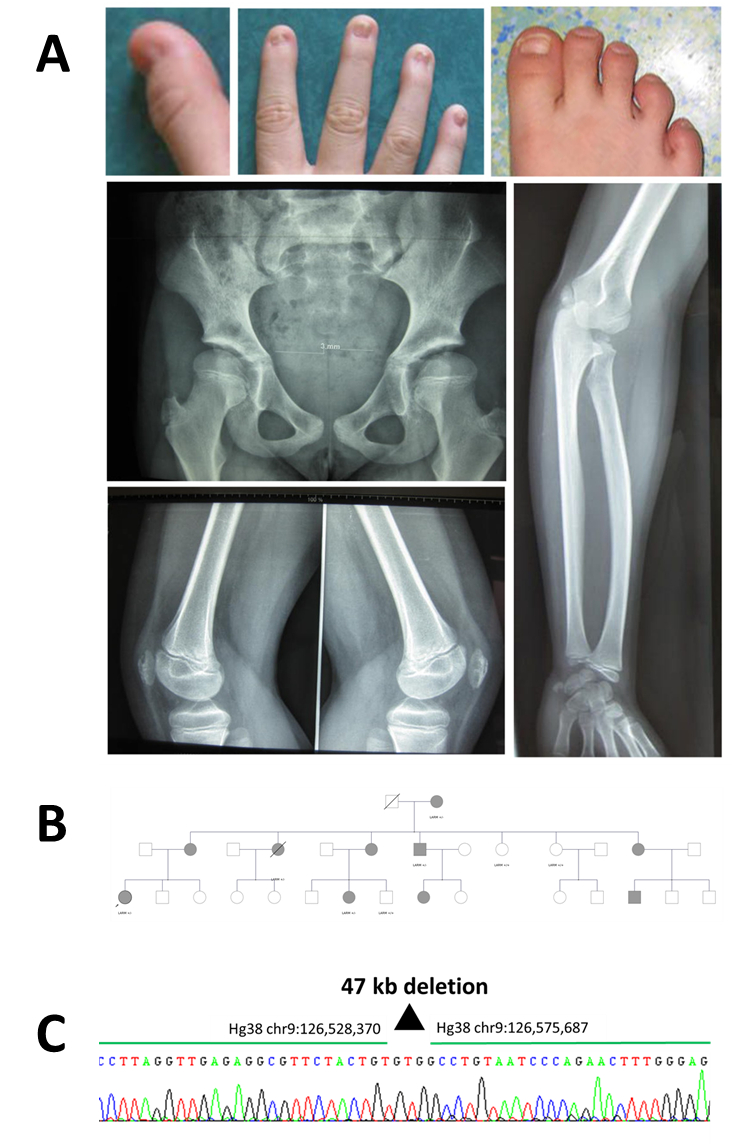


A. Clinical pictures and X-rays of Patient 1, showing dystrophic hand nails, radial head dysplasia with posterior subluxation, hypoplastic patellae and iliac horns, typical for NPS syndrome. B. Pedigree of the family, showing autosomal dominant mode of inheritance of the Nail-Patella syndrome. C. Sanger sequencing confirming the 47 kb deletion comprising LARM1 and LARM2.

**Supplementary Figure 2**


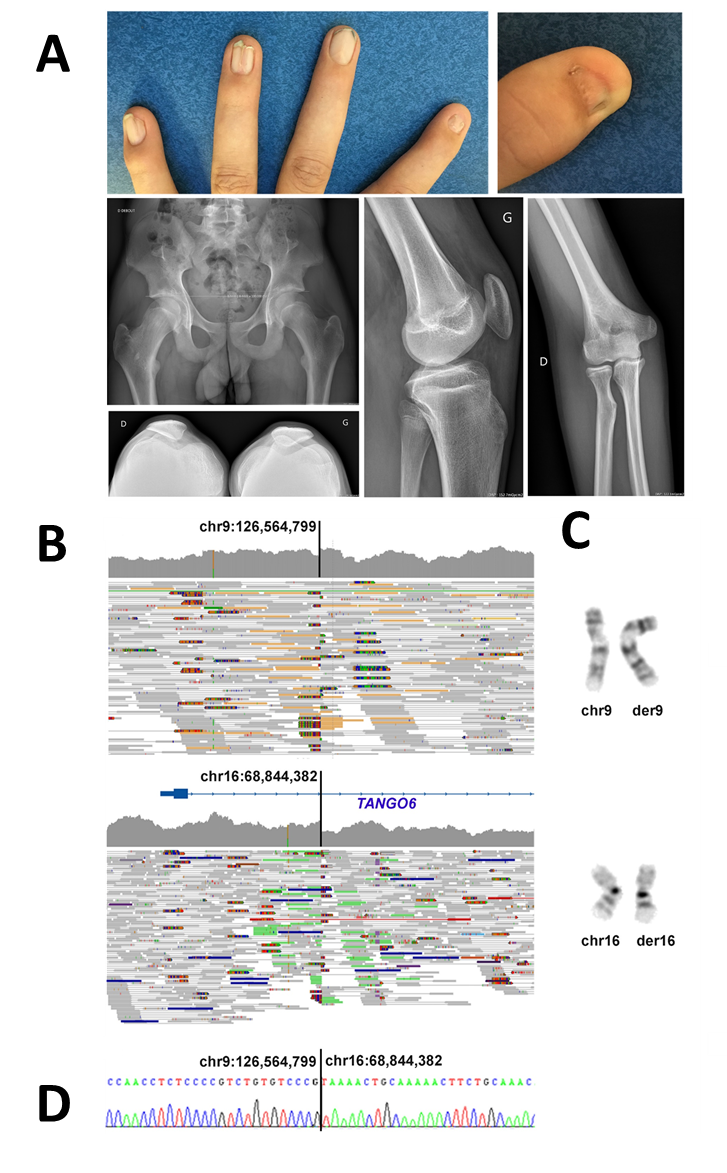


A. Clinical pictures and X-rays of Patient 2 showing dystrophic hand nails, radial head dysplasia, patella dysplasia and iliac horns, typical for NPS syndrome. B. Examination of the *LMX1B* locus using IGV browser showing split-reads, unmasking a reciprocal translocation t(9;16)(q33.3;q22.1). The 9q33.3 breakpoint (chr9:126,564,799(hg38)) is located in the non-coding region in between *LMX1B* and LARM1/2, while the 16q22.1 breakpoint (chr16:68,844,382(hg38)) interrupts the *TANGO6* gene. C. Confirmation of the t(9;16) translocation by karyotyping on leucocytes. D. Confirmation of the t(9;16) translocation breakpoints by Sanger sequencing.

**Supplementary Figure 3**


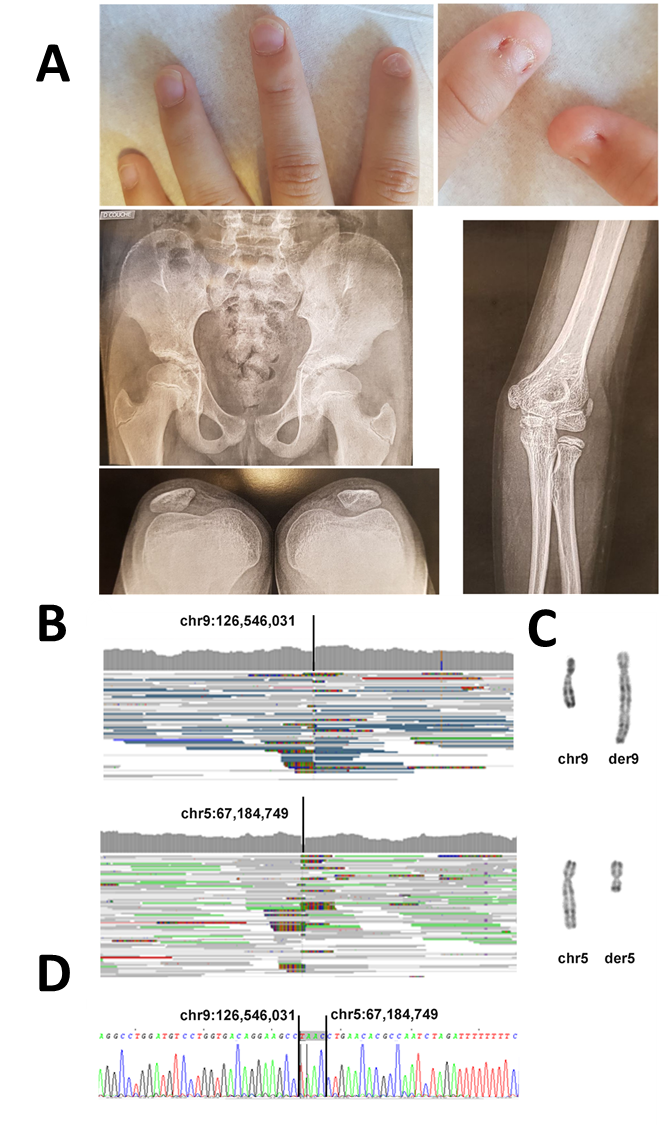


A. Clinical pictures and X-rays of Patient 3 showing dystrophic hand nails, radial head dysplasia, patella dysplasia and iliac horns, typical for NPS syndrome. B. Examination of the *LMX1B* locus using IGV browser showing split-reads, unmasking a reciprocal translocation t(5;9)(q12.3;q33.3). The 9q33.3 breakpoint (chr9:126,546,031(hg38)) is located in the non-coding region in between *LMX1B* and LARM1/2, while the 5q breakpoint (chr5:67,184,749(hg38)) interrupts the *CD180* gene. C. Confirmation of the t(5;9) translocation by karyotyping on leucocytes. D. Confirmation of the t(5;9) translocation breakpoints by Sanger sequencing.

**Supplementary Figure 4**


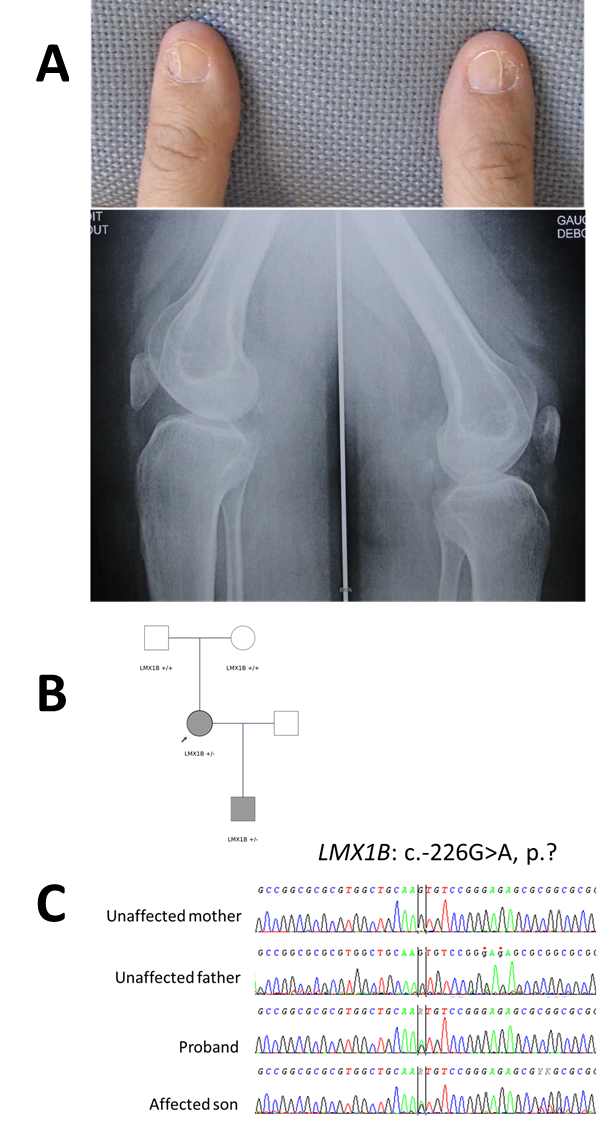


A. Clinical pictures and X-rays of Patient 4 showing dystrophic hand nails and patella hypoplasia, typical for NPS syndrome. B. Pedigree of the family. C. Sanger sequencing confirmation of the heterozygous variant NC_000009.12:g.126614224G>A; NM_001174147.2:c.-226G>A; p.?, located in the 5’UTR of LMX1B gene, which occurred *de novo* in the proband and segregates in her affected son.
